# Neuroprotective role of PKR in a model of neurodegeneration due to mild impairment of oxidative metabolism

**DOI:** 10.1186/1750-1326-8-S1-P31

**Published:** 2013-09-13

**Authors:** François Mouton-Liger, Anne-Sophie Rebillat, Sarah Gourmaud, Audrey Leguen, Claire Paquet, Laurent Pradier, Thomas Rooney, Jacques Hugon

**Affiliations:** 1 Inserm UMR-S839, Paris, France; 2 Hopital Lariboisière APHP, Paris, France; 3 Sanofi, Chilly-Mazarin, France

## Background

Abnormalities in oxidative metabolism, inflammation, and selective neuronal death are described features of Alzheimer’s disease (AD) pathology. Brain thiamine-dependent enzymes play an important role in energy metabolism and display reduced activity in AD. Thiamine (vitamin B1) deficiency (TD) induces regionally selective neuronal death in animal and human brains. TD-induced neuronal loss is associated with a mild and chronic impairment of oxidative metabolism as well as inflammatory responses with cytokine production. These features make the TD model amenable to investigate the cellular mechanisms of neurodegeneration. PKR (double stranded-RNA dependent protein kinase) is activated by Aß_1-42_, and by various cellular stresses including oxidative stress. Once activated, PKR acts as a pro-apoptotic kinase and negatively controls the initial step of protein translation by the elF2α phosphorylation, leading to an increase of BACE1 translation. In addition, it has been demonstrated that TD promotes PKR activation.

## Learning objective

The goal of this study was to use the TD model in mice to assess *in vivo* the involvement of PKR in the neuronal death, inflammation and in the neuropathological mechanisms of AD. All results of this TD model are compared with those obtained in controls (saline injected).

## Methods

C57BL/6J mice, housed in a controlled environment, have been fed either a control diet or a thiamine deficient diet *ad libitum.* TD animals also received a daily intraperitoneal injection of a thiamine antagonist, pyrithiamine hydrobromide for 10 days while control animals will be injected with saline.

## Results

Our results showed that the PKR-elF2α pathway, BACE1 levels and Aβ production are increased in the thalamus and cerebellum of the TD model. These findings are associated with microglial activation and neuronal death. Performance on the rotarod task also declines in the animal TD model in a highly reproducible manner.

## Conclusion

Our findings suggest a role *in vivo* of the kinase PKR in neuronal degeneration linked to oxidative metabolism impairment.

